# CT imaging findings following treatment with combination SBRT and chemotherapy versus stand-alone chemotherapy for locally advanced pancreatic adenocarcinoma

**DOI:** 10.1007/s00261-025-05066-9

**Published:** 2025-06-19

**Authors:** Vishaal Gudla, Abraham F. Bezuidenhout, Mishal Mendiratta-Lala, Olga R. Brook, Vassilios Raptopoulos, Matthew J. Abrams, Alexander Brook, Bettina Siewert

**Affiliations:** 1 Beth Israel Deaconess Medical Center, Boston, USA; 2 University of Michigan-Ann Arbor, Ann Arbor, USA

**Keywords:** Pancreatic adenocarcinoma, Stereotactic body radiotherapy (SBRT), Locally advanced pancreatic cancer (LAPC)

## Abstract

**Purpose:**

To evaluate imaging findings of patients with locally advanced pancreatic cancer (LAPC) following treatment with combination stereotactic body radiotherapy (SBRT) and chemotherapy versus stand-alone chemotherapy.

**Methods:**

This retrospective study included patients with LAPC who received combination SBRT/chemotherapy versus those that received stand-alone chemotherapy from 2005 to 2018. Comparisons were made pre-treatment and at four standardized post-treatment intervals (1 month, 3–6 months, 7–12 months, greater than 12 months) in patients without disease progression. Imaging variables included degree of vascular involvement graded on a standardized scale and peripancreatic fat stranding. A p-value < 0.05 was considered significant.

**Results:**

A total of 96 patients were included, 64 patients (37 men; mean age, 68 ± 11 years) treated with SBRT/chemotherapy and 32 patients (17 men; mean age, 69 ± 10 years) treated with stand-alone chemotherapy. Increased vascular involvement over time in the absence of disease progression was significantly higher in the SBRT/chemotherapy group (17%) versus the stand-alone chemotherapy group (9%), *p* = 0.004 (95% CI 2–12%). Peripancreatic fat stranding increased over time in the SBRT/chemotherapy group being present in 29/64 (45%) patients on the pre-treatment computed tomography (CT) versus 55/64 (86%) patients on the last recorded CT, *p* < 0.001 (McNemar OR = 14, 95% CI 3.3–58.8). No significant change in peripancreatic fat stranding over time was noted in the stand-alone chemotherapy group being present in 9/32 (28%) patients on the pre-treatment CT and 7/32 (22%) patients on the last imaging recorded, *p* = 0.45 (McNemar OR = 2, 95% CI 0.4–10.9).

**Conclusions:**

Increased vascular involvement and peripancreatic fat stranding over time in LAPC patients treated with combination SBRT/chemotherapy should be a potential anticipated treatment-related effect, not necessarily indicating disease progression.

## Introduction

Pancreatic ductal adenocarcinoma is the third leading cause of cancer-related death in the United States, following lung and colon cancer, and is expected to become the 2nd leading cause by 2030 [1]. While other cancers have seen a decline in mortalities over the last few decades, the mortality rate of pancreatic adenocarcinoma has remained relatively stable [1].

Resection followed by adjuvant therapy remains the standard of care for resectable pancreatic adenocarcinoma. Neoadjuvant therapy has become increasingly common for high risk patients with major goals including reducing tumor volume before surgery, reducing rates of positive resection margin, reducing rates of positive lymph nodes, treating imaging occult micrometastases, and attempting to downstage a tumor to resectable status [2, 3]. Additionally, stereotactic body radiotherapy (SBRT) has shown promise as a potential alternative to conventional external beam radiotherapy for the treatment of borderline resectable and locally advanced pancreatic cancer (LAPC). It has been shown to offer several benefits including higher biologic equivalent dose, reduced volume of irradiated healthy tissue, shortened treatment time, and reduced rates of local recurrence [4–12].

Computed tomography (CT) remains the gold standard for staging pancreatic adenocarcinoma both before and after treatment. Despite the increasing use of neoadjuvant therapy, accurate disease assessment during and following neoadjuvant therapy has remained a diagnostic challenge [2]. In particular, little is known about the imaging appearance of LAPC patients treated with combination SBRT and chemotherapy (SBRT/chemotherapy) as this is a relatively new treatment option. The major limitation within the current literature on imaging assessment following SBRT is the lack of sequential and standardized follow up [2–5].This information is of clinical importance as knowledge concerning the imaging evolution of LAPC patients treated with combination SBRT/chemotherapy will aid in accurately assessing disease during and post-treatment.

The purpose of this study was to characterize the CT imaging features of LAPC patients treated with SBRT/chemotherapy versus those treated with stand-alone chemotherapy.

## Methods

### Patient sample

This Health Insurance Portability and Accountability Act (HIPAA) compliant retrospective study was approved by the institutional review board with a waiver of the requirement for written informed consent and followed Standards for Reporting of Diagnostic Accuracy Studies (STARD) reporting guidelines.

The radiology information system was searched for the records of patients older than 18 years who underwent multiphasic pancreatic CT angiography with a clinical indication of non-resectable LAPC between January 1, 2005 and December 31, 2018. Treatment protocols for all identified patients meeting this criterion were determined and those who received CyberKnife (Accuracy Inc., Sunnyvale, CA) SBRT and chemotherapy (SBRT/chemotherapy) were deemed the treatment group while those that received stand-alone chemotherapy were deemed the control group. Patients that did not undergo neoadjuvant treatment at our institution and/or those without follow up CT examinations for imaging assessment were excluded.

### CT examinations

Patients underwent contrast-enhanced abdominal CT with a 64- or 128 multidetector computer tomography system (LightSpeed VCT, GE Healthcare; Aquilion, Toshiba America Medical Systems). The tube voltage was approximately 120 peak kilovoltage (kVp), and the tube current was determined by the automatic exposure control. Intravenous contrast medium was administered at a flow rate of 3–5 mL/s. Examinations were performed with multiphase pancreatic protocol (pancreatic phase 30–40 s and portal venous phase 70 s after injection). Images were reconstructed at a slice thickness of 2.5 mm or less in the axial, coronal, and sagittal planes.

### CT interpretation

**Fig. 1:**
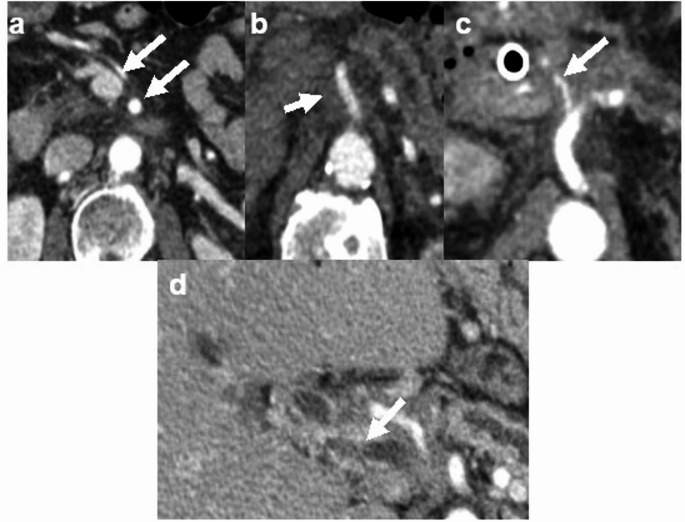
Axial contrast enhanced CT images depict grading of vascular involvement. **a** No vascular involvement of superior mesenteric vessels, grade 0 (arrows). **b** Encasement of the superior mesenteric artery without stenosis, grade 1 (arrow). **c** Encasement with stenosis of the common hepatic artery, grade 2 (arrow). **d** Encasement and occlusion of the main portal vein, grade 3 (arrow)

The CT examinations were evaluated in consensus by two investigators blinded to clinical and treatment data (VG, a fellowship-trained abdominal radiologist with 2 years of post-training experience including 1 year of pancreaticobiliary multidisciplinary conference participation and AB, a fellowship-trained abdominal radiologist with 8 years of post-training experience including 6 years of pancreaticobiliary multidisciplinary conference participation). For each patient, CT examinations pre-treatment and at four subsequent standardized time intervals were reviewed (1 month, 3–6 months, 7–12 months, greater than 12 months). At each time point, degree of vascular involvement and the presence of peripancreatic fat stranding were recorded. Degree of vascular involvement was recorded for eight vessels: portal vein, superior mesenteric vein, splenic vein, left renal vein, celiac artery, common hepatic artery (including replaced vessels), superior mesenteric artery and splenic artery. A total of 512 individual vessels (8 vessels in each of the 64 patients) were assessed in the combination SBRT/chemotherapy group and 256 individual vessels (8 vessels in each of the 32 patients) were assessed in the stand-alone chemotherapy group. Vascular involvement was graded on a four-point scale as follows: 0—no involvement/less than 180-degree abutment, 1—encasement without stenosis or occlusion, 2—encasement with stenosis, 3—vessel occlusion (Fig. 1).

### Neoadjuvant therapy

Chemotherapy regimens for patients in this study generally reflected the evolving standard of care over the study period. Prior to 2010, gemcitabine-based regimens, often administered in combination with other agents, were the predominant approach. Following 2010, folinic acid, fluorouracil, irinotecan and oxaliplatin (FOLFIRINOX) became the preferred regimen in patients with preserved performance status, whereas gemcitabine plus nab-paclitaxel was more frequently utilized in patients with limited functional reserve. The choice of chemotherapeutic agents were made independent of potential future SBRT use or not.

For patients who received SBRT, clinical target volume (CTV) included the gross tumor volume (GTV) and involved the vasculature. The planning target volume (PTV) was a uniform 3 mm expansion of the PTV. The PTV was cropped out of critical structures by the treating radiation oncologist as needed to meet dose constraints. In the majority of cases, the PTV received 27 Grays in 3 fractions (institutional standard). The interdigitation of SBRT varied slightly from patient to patient, predominantly occurring during the 1st month following induction chemotherapy.

### Disease progression

Patients were assessed for disease progression at each follow up CT examination. Disease progression was determined by a combination of clinical assessment (including patient performance status and clinical symptoms) and trends in Cancer Antigen 19-9 (CA 19-9) serology as assessed at multidisciplinary tumor board and documented in the electronic medical record. For both treatment and control groups, tumor marker trends over time were interrogated at each time point, and those in whom an increase was noted from baseline were defined as having biochemical progression at the time point where CA 19-9 elevation was first documented. Per institutional standard, a CA 19 -9 increase of 20% from baseline was considered biochemical progression. The only exception to this criterion was among SBRT patients during the first treatment interval (pre-treatment to one-month post-treatment), when a transient increase in CA 19-9 was tolerated and presumed secondary to radiation effects from SBRT. Such patients were only allowed to remain within the dataset if their tumor marker values returned to baseline or below at the second standardized time interval (3–6 months); failure to do so led to exclusion.

Among those patients in whom progression was identified, partial exclusion was performed wherein data at and beyond the time point when progression was initially suspected was excluded, leaving only pre-progression data. For explanatory purpose, a patient in whom progression was suspected by CA 19-9 elevation from baseline at nine months post-treatment would have all data from the nine-month post-treatment time point and beyond excluded from evaluation, with the pre-treatment, one-month post-treatment, and three-to-six month post-treatment data remaining within the dataset.

### Statistical analysis

Data was summarized with descriptive statistics. Unpaired *t*-test was used to compare ages. Chi-squared test was used to compare distributions of gender. A mixed-effects logistic regression model with random effects for patient and fixed effects for vessels were used to analyze vascular involvement over time. Chi-squared and McNemar test were used to compare prevalence of fat stranding in different subgroups. Mann–Whitney test was used to compare length of follow-up. *P* < 0.05 was considered statistically significant. Computations were performed with Matlab software (version 9.9, MathWorks).

## Results

### Patient sample

**Fig. 2:**
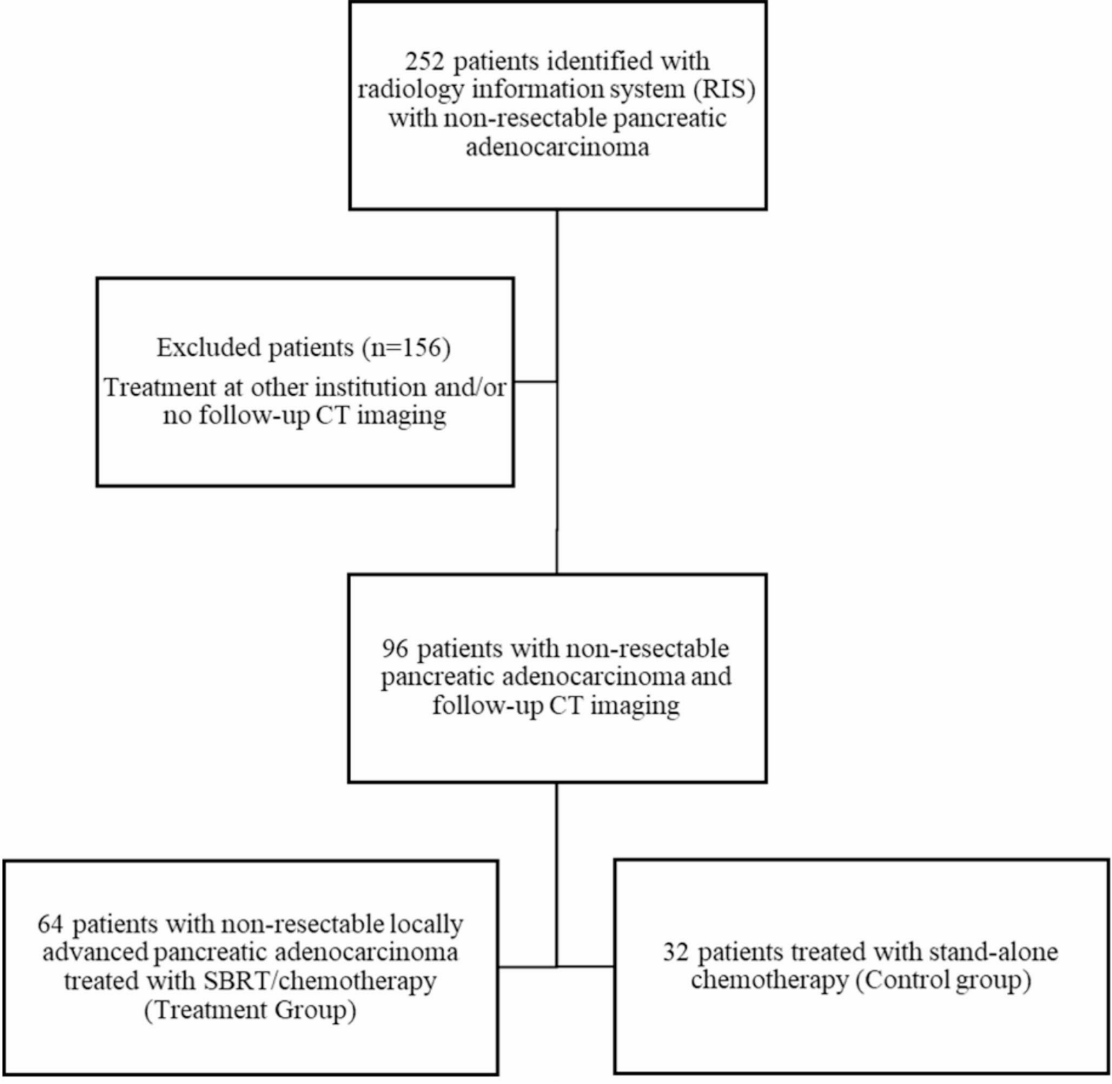
Flow diagram shows steps in patient selection

The initial search identified 252 patients with LAPC. 156 patients were excluded because of receiving treatment at other institutions and/or no available subsequent CT examinations. A total of 96 patients formed the final study sample with 64 patients (37 men; mean age, 68 ± 11 [standard deviation] years) treated with combination SBRT/chemotherapy and 32 patients (17 men; mean age, 69 ± 10 years) treated with stand-alone chemotherapy. Figure 2 shows the flow of patient selection. There was no significant difference in age or sex between the two groups (*p* = 0.75 and 0.66 respectively). Median length of follow up for the combination SBRT/chemotherapy group was 237 days (Interquartile range [IQR] 114–395) and for the stand-alone chemotherapy was 102 days (IQR 44–293), *p* = 0.02.

### Vascular involvement

**Fig. 3:**
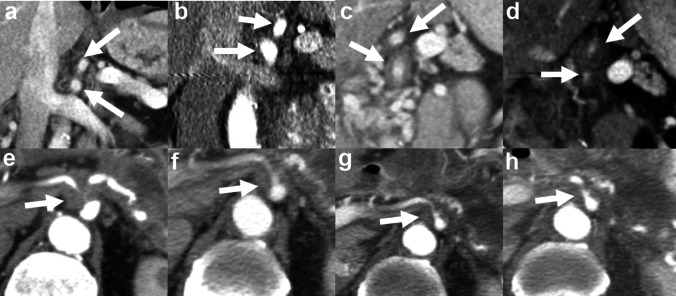
**a**–**d** Coronal contrast enhanced CT images of a patient treated with combination SBRT/chemotherapy over time with arrows noting changes in the celiac and superior mesenteric arteries, in the absence of disease progression. No vascular involvement of the celiac and superior mesenteric arteries pre-treatment (**a**) and 1 month post-treatment (**b**); encasement without stenosis of these vessels 6 months post-treatment (**c**); encasement with stenosis of these vessels 12 months post-treatment (**d**). **e**–**h** Axial contrast enhanced CT images of a patient treated with stand-alone chemotherapy over time with arrows noting the celiac artery. There is encasement of the celiac artery with stenosis pre-treatment (**e**) which does not change at 1 month (**f**), 6 month (**g**) and 12 month (**h**) post-treatment intervals

A mixed-effects logistic regression model showed that increased vascular involvement over time was significantly higher in the SBRT/chemotherapy group (88/512 [17%] of evaluated vessels) versus the stand-alone chemotherapy group (24/256 [9%] of evaluated vessels), in the absence of disease progression, *p* = 0.004 (95% CI 2–12%). Vessels that most frequently demonstrated increased involvement in the SBRT/chemotherapy group were the common hepatic artery (15/64 [23.4%] patients), celiac artery (13/64 [20.3%] patients), and superior mesenteric vein (13/64 [20.3%] patients). Table 1 summarizes this data. Figure 3 demonstrates examples of vascular involvement over time in each group.

**Table 1:** Comparison of increased vascular involvement of individual vessels over time in the combination sbrt/chemotherapy group versus the stand-alone chemotherapy group, in the absence of disease progression

Vessel	Increase in involvement	
	SBRT/chemotherapy (*n* = 64)^a^	Chemotherapy (*n* = 32)^a^
Portal vein	7 (10.9%)	4 (12.5%)
Splenic vein	9 (14.1%)	2 (6.2%)
Superior mesenteric vein	13 (20.3%)	4 (12.5%)
Left renal vein	8 (12.5%)	2 (6.2%)
Common hepatic artery	15 (23.4%)	3 (9.4%)
Celiac artery	13 (20.3%)	3 (9.4%)
Superior mesenteric artery	11 (17.2%)	4 (12.5%)
Splenic artery	12 (18.8%)	2 (6.2%)

^a^Reported as number of patients with increased involvement (percentage of group)

### Peripancreatic fat stranding

Peripancreatic fat stranding increased over time within the SBRT/chemotherapy group, with peripancreatic fat stranding being present on the pre-treatment CT of 29/64 (45%) patients increasing to 55/64 (86%) patients on the last CT recorded, *p* < 0.001 (McNemar OR = 14, 95% CI 3.3–58.8). No significant change in peripancreatic fat stranding was noted over time within stand-alone chemotherapy group, with peripancreatic fat stranding being present on the pre-treatment CT of 9/32 (28%) patients and 7/32 (22%) patients on the last CT recorded, *p* = 0.45 (McNemar OR = 2, 95% CI 0.4–10.9).

## Discussion

Imaging plays a crucial role in staging and assessing treatment response in patients with LAPC, especially with the increasing use of neoadjuvant chemoradiation. In this study, we evaluated the imaging findings and how they change over time in patients with LAPC treated with combination SBRT/chemotherapy versus stand-alone chemotherapy, in the absence of disease progression. Increased vascular involvement over serial CT studies was noted with increasing frequency in the SBRT/chemotherapy group (17%) versus the stand-alone chemotherapy group (9%) in the absence of disease progression. Additionally, peripancreatic fat stranding increased over time in patients treated with SBRT/chemotherapy being present on the initial CT study in 29/64 (45%) versus 55/64 (86%) patients’ last recorded CT study, in the absence of disease progression. Peripancreatic fat stranding did not change significantly over serial CT studies in patients treated with stand-alone chemotherapy. These findings are likely secondary to the fibrotic response induced by combination SBRT/chemotherapy and should not be taken at face value as disease progression [14]. SBRT allows for high, localized radiation doses which prompt an inflammatory cascade causing the differentiation of fibroblasts to myofibroblasts. In turn, there is increased production of extracellular matrix components leading to dense fibrotic tissue at the site of injury [13, 14]. In the setting of pancreatic adenocarcinoma, fibrotic tissue has similar attenuation value to scirrhous tumor tissue which complicates imaging interpretation.

Several prior studies have assessed the imaging appearance of LAPC following neoadjuvant chemoradiotherapy and current literature underscores the unreliability of CT staging of LAPC following neoadjuvant chemoradiation [2, 15]. However, the current study is unique in that we specifically studied patients treated with combination SBRT/chemotherapy rather than conventional radiation therapy and chemotherapy. While the imaging appearance following SBRT/chemotherapy of other cancers, such as liver and lung, have been studied, little is known about the imaging appearance of LAPC following SBRT/chemotherapy [16, 17]. Further knowledge of the expected imaging findings of LAPC during and following SBRT/chemotherapy is important for radiologists when interpreting these imaging studies.

A limitation of this study was its retrospective design and single center setting. Patients without follow up imaging were excluded from the study, leading to potential selection bias. The median length of follow-up in the SBRT/chemotherapy group was higher than that of the group treated with stand-alone chemotherapy, however this was due to decreased survival periods for patients in the stand-alone chemotherapy group as SBRT is typically employed in patients with smaller tumors and localized or oligo-metastatic disease. Another potential limitation is the presence of unknown co-morbidities and concurrent medication use which could alter the response to radiation therapy. Pathologic correlation for disease progression was not available in many patients, as it is not the standard of care to subject these patients to invasive procedures, when clinical factors and CA19-9 serology utilized at multidisciplinary tumor board is highly suggestive of disease progression. Prospective studies incorporating radiologic-pathologic correlation could provide further insight into the findings seen following SBRT/chemotherapy.

In conclusion, increasing vascular involvement and peripancreatic fat stranding in patients with LAPC treated with combination SBRT/chemotherapy may be an anticipated treatment effect and does not definitively indicate disease progression or treatment failure. Radiologists should be aware of this imaging manifestation, provide an appropriate differential diagnosis, and prompt multidisciplinary management. The ultimate treatment plan for a patient should be made with the integration of clinical, laboratory and radiologic data.

## Data Availability

Raw data cannot be shared publicly to maintain patient confidentiality. Relevant data is provided within the manuscript.
